# Collinear Motion Strengthens Local Context in Visual Detection

**DOI:** 10.1177/2041669520961125

**Published:** 2020-10-21

**Authors:** Massimo Girelli

**Affiliations:** Department of Neuroscience, 19051 University of Verona, Verona, Italy

**Keywords:** context effect, collinear motion, long-range connections, corticocortical feedback, corticothalamic feedback

## Abstract

Detection of elongated objects in the visual scene can be improved by additional elements flanking the object on the collinear axis. This is the collinear context effect (CE) and is represented in the long-range horizontal connection plexus in V1. The aim of this study was to test whether the visual collinear motion can improve the CE. In the three experiments of this study, the flank was presented with different types of motion. In particular, the collinear motion aligned with the longitudinal axis of the to-be-detected object: toward or away from it, and the orthogonal motion with a direction perpendicular to the collinear axis. Only collinear motion toward the target showed a robust and replicable empowerment of the CE. This dynamic modulation of the CE likely is implemented in the long-range horizontal connection plexus in V1, but, given that in addition it conveys the time information of motion, there must be a direct feedback in V1 from higher visual areas where motion perception is implemented, such as Middle Temporal (MT). Elongated visual objects moving along their longitudinal axis favor a propagation of activation in front of them via a network of interconnected units that allows the visual system to predict future positions of relevant items in the visual scene.

Object detection is greatly influenced by the static or dynamic context of the visual scene. Gestalt psychologists suggested that similarity, proximity, and common fate can integrate a contour of an object to increase its saliency and to stand out against a background (Wertheimer, 1912). The well-known context effect (CE) allows better detection of simple bars presented against a homogeneous background when they are flanked by a high-contrast similar bar. The flank, though, must be aligned along the collinear axis of the to-be-detected bar and close enough to be integrated in an elongated border (Polat & Sagi, 1993, 1994). Neurophysiological evidence supports the idea that the CE is represented in primary visual cortex of primates where the response elicited by a simple bar, falling into the V1 neuron’s receptive field, was potentiated by the presence of a collinear flank outside the receptive field (Kapadia et al., 1995). When this flank presented small deviations from the collinear axis of the target or it was not close enough to the target or, finally, it was replaced by a T-shaped flank that interrupted the good continuation of the border, the CE disappeared. The CE was observed in orientation-selective neurons in superficial layers of V1, connected via a plexus of long-range horizontal connections (LRHC; Gilbert et al., 1996; Kapadia et al., 1995), with the same orientation selectivity and lying along a collinear path. Recently, recordings in primate V1 suggested that the CE might result, at least in part, from the collinear elongation of the receptive field of the V1 population responses in the retinotopic orientation representation (Michel et al., 2013).

With a long border, the CE is repeated indefinitely resembling the trajectory of an elongated stimulus moving in a direction collinear to its longitudinal axis. Moreover, the CE might propagate in front of the moving stimulus as a preactivation of the most likely path assuming that, in the real world, moving objects usually keep moving in the same direction (Anstis & Ramachandran, 1987; Ramachandran & Anstis, 1983). Collinear motion, therefore, predicts the presence of an object in a future position in space when it is in a close spatiotemporal proximity (Jachim et al., 2017; Morgan & Chubb, 1999; Verghese & McKee, 2002). Delay lines in Grzywacz’s theory of temporal coherence for motion detection (Grzywacz et al. 1995; Verghese et al., 1999) and visual momentum both make and implement the assumption that moving objects usually follow a straight or quasistraight path. According to Ramachandran and Anstis, the perceptual visual system, once a direction of motion is recognized, is inclined to see the same direction of motion (Ramachandran & Anstis, 1983). This sort of motion CE must rely on higher order cerebral areas such as Middle Temporal (MT), sensitive to motion, which can modulate the V1 motion units via feedback (see ahead in the Introduction section) and can get quickly the motion signals by thalamocortical pathway directly from lateral geniculate nucleus (LGN; Sincich, 2004; Zeki, 2015). Therefore, an elongated moving stimulus might serve as a flank for itself in that it will preactivate the motion path ahead of its present position as a bow wave in front of a boat. A possible hint of this motion CE was provided years ago by Verghese and coworkers in an experiment with moving dots in which the observers had to report which of two intervals presented a coherent moving stimulus. The moving stimuli were triplets of dots in coherent motion (resembling elongated objects) that could move either along a trajectory parallel to their orientation (collinear motion) or along a trajectory vertical to their orientation (orthogonal motion). Results showed that a triplet in the collinear motion condition was much more detectable than a triplet in the orthogonal motion condition (Verghese et al., 2000). These authors proposed that “ . . . trajectory is the motion-equivalent of the static contour system and that it is likely coded by a combination of signals from the primary motion units stimulated along the direction of motion (Verghese et al., 2000; p. 1533).” On the contrary, according to a model put forward by Geisler (1999), the collinear motion provided only the orientation signal through static detectors, while the motion signal was still guaranteed by motion detectors orthogonally oriented with respect to the trajectory of motion, resembling the speed lines (Burr, 2000; Burr & Ross, 2002). Very recently, I also provided evidence of collinear motion advantage in an experiment using the Poggendorff illusion: A collinearly moving bar yielded the most accurate alignment judgment and almost cancelled the well-known misalignment produced by the illusion (Girelli, 2014), while an orthogonally moving bar or a simple dot did not and showed large illusory misalignments. In a previous study by Watamaniuk (2005), the same visual illusion and moving stimuli were used. In particular, he replaced the oblique segments that appear to form a line behind an occluder of the typical Poggendorff illusion with dots moving along a rectilinear path. He also used a mixed condition in which only one segment was replaced by a moving dot. The results showed that the advantage in the alignment judgment of a moving stimulus, with respect to the static segment, was obtained only when the first segment was replaced by a dot moving toward the occluder. Although there might be undoubtedly some similarities with my 2014 study, there is one crucial difference: I used a moving bar instead of a simple dot. Therefore, in my study, the stimulus could move along a collinear direction of motion, either toward or away from a target stimulus, while Watamaniuk’s dot stimulus could not because a dot does not have a collinear axis and therefore cannot address the collinear motion advantage.

The CE of static stimuli, therefore, could be further potentiated by collinear motion, and the LRHC plexus in V1, representing both the temporal and the orientation signals, might be the best candidate to provide the neural basis of the temporal coherence theory or of the correspondence problem in visual motion (Anstis, 1980; Grzywacz et al., 1995; Marr, 1982; Ullman, 1979). Both motion and orientation signals develop in time as a unique signal of motion trajectory in V1. Roughly after 200 ms, the orientation signal represents the motion trajectory collinear to the orientation (Jancke, 2000). Collinearly moving bars, therefore, might activate, along the motion path, V1 orientation-selective neurons as well as V1 motion-selective neurons which modulates each other. This mixed orientation-motion signal, given its latency of 200 ms, is likely to be accomplished via feedback connections from MT to Layer 6 of V1, via local LRHC connections in V1 (Gilbert et al., 1996) and downstream from V1 to the LGN (Cudeiro & Sillito, 2006; Sillito et al., 2006). Orientation, position, and time information are provided, therefore, in an interconnected network of orientation-selective columns with the same orientation preference aligned along the collinear motion path, connected by the LHRC, and carrying over the time information. The overall motion signal and in particular the direction of motion is likely to be extracted in MT where more information from different parts of the visual scene converges in single units to represent one direction. Once this information is processed in MT, it can modulate downstream, via feedback pathways, V1 activity, and in particular the processing of the columns showing the same orientation preference, aligned along the collinear path and connected via the LHRC plexus.

The aim of this study was to provide experimental evidence of an advantage in object detection, through the CE, of collinearly moving stimuli with respect to orthogonally moving or static stimuli. In the first experiment, the CE produced by collinear motion was compared with that produced by orthogonal motion and to absence of motion. In the second experiment, a well-known ability of a horizontal Border, placed between the target and the flank, in nullifying the CE was tested with the collinear motion. Finally, in the third experiment, the direction of collinear motion, toward or away from the target, was tested. In the three experiments, the predictions based on the experimental hypotheses tested were threefold: First, collinear motion should generate a more robust CE with respect to orthogonal motion and absence of motion; second, collinear motion should overcome the action of nullifying the CE by the horizontal Border; and third, a direction of collinear motion toward the target should generate an additional advantage in the CE with respect to a collinear motion heading away from the target. This final prediction resembled an advantage of toward versus away direction of motion in propagating the motion signal onto a crucial element reported in 2005 by Watamaniuk (2005), although he did not use an elongated object but a simple dot; therefore, in that study, motion cannot be defined as collinear.

## Methods

### Participants

For each of the three experiments of the study, two naïve participants and the author performed in the tasks (mean age = 37). They were all right-handed, neurologically intact, and had normal or corrected-to-normal vision. They signed a consent form to participate in the experiment carried out according to the World Medical Association Declaration of Helsinki published on the website of the *Journal of American Medical Association* in 2013 and approved by the local ethical Committee of the University of Verona.

### Stimuli and Task

The stimuli were displayed onto a CRT-60 Hz 17″ Philips monitor (dot pitch 0.25 mm—dot pitch [horizontal] 0.21 mm) placed at a distance of 114 cm from the participants. A chin rest was used to limit head movements. The participants fixated a bright small white cross (luminance 90 cd/m^2^) in the center of the display on a gray background (luminance 0.4 cd/m^2^).

**Figure 1.**
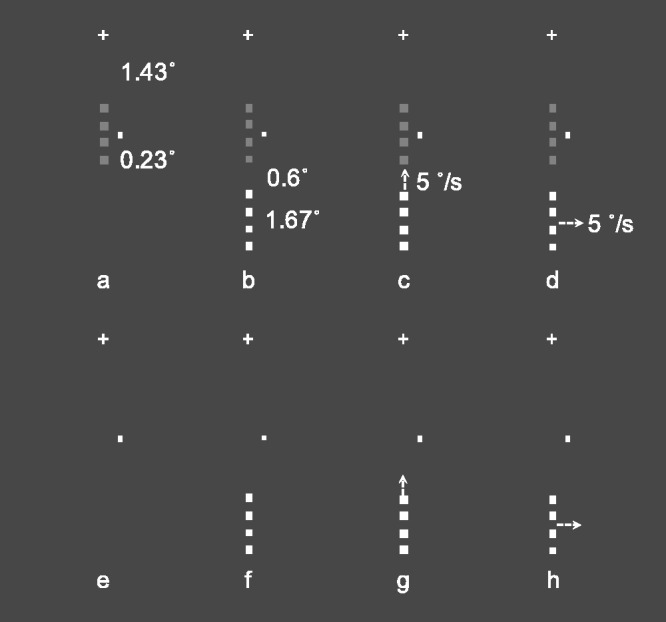
Stimuli in Experiment 1. Target-present (A) and target-absent (E) trials used as baseline to evaluate the context effect. Target-present trials (at one of six contrasts) (B, C, and D) and target-absent trials (F, G, and H) in the three experimental conditions. In detail: Static (ST) condition: target-present (B), target-absent (F); Collinear Motion (CM) condition: target-present (C), target-absent (G); Orthogonal Motion (OM) condition: target-present (D), target-absent (H). The small white cross above each panel represents the fixation point, and the small rectangle to the right of the Target, the attentional cue denominated Dot, was present in the display at all times.

The basic stimulus used in all experiments was made of four squares (side 0.23°) perfectly aligned and spaced, edge-to-edge, at 0.25° to cover a total length of 1.67°. This basic stimulus was aligned to the vertical meridian and placed, with the upper side of the first square element at 1.43° from the fixation cross in the lower visual field. In this spatial position, this stimulus was denominated the Target, and its luminance was varied according to the constant stimuli method (see Figure 1A). The Target was stationary at all times in all the experimental conditions of the three experiments of the study. A little white rectangle (0.06°), denominated Dot (luminance 90 cd/m^2^), was placed halfway the length of this basic stimulus, 0.3° to the right and served as an attentional cue for the observer to keep attention on that area, in particular, when the target was presented at low contrasts. This stationary attentional cue was always present in all trials of all conditions (see Figure 1A and E). Along the vertical meridian, at 0.6° below the lower side of the lowest squared element of the Target, another basic stimulus, denominated Flank, was presented in white (luminance 90 cd/m^2^) extending in the lower visual field for 1.67° (see Figure 1F). The Flank was the only element moving in the visual displays in all experiments (see the following for the methodological details for each experiment). The choice of this particular stimulus, made of four identical elements in a particular configuration, was mandatory to have the same amount of local motion signal in the collinear and orthogonal motion directions. That is, in both directions, there were always four straight edges of the same length and moving for the very same amount of space: In case of a solid bar, on the contrary, the orthogonal motion direction would have shown a larger moving edge, with respect to the collinear motion direction. All the stimuli were presented for 50 ms.

In the three experiments, the observers were required to signal the presence or absence of the target in a two-alternative forced choice by pressing two different buttons with the index finger of the two hands on a response pad: The response-to-key mapping and the hand-to-key mapping with respect to the presence of the target was counterbalanced across the experimental blocks for each observer. Therefore, there were two main categories of trials: target-present and target-absent; within the first category, there were Target and Target+Flank stimuli (see Figure 1A and B, respectively), while in the target-absent trial category, there were Dot and Flank stimuli (see Figure 1E and F, respectively).

The stimuli described earlier were presented in different experimental conditions in the three experiments as follows.

### Experiment 1

In target-present trials, in one condition, the flank was presented motionless: Static (ST) condition (Figure 1A and B); in a different condition, the Flank moved collinearly along the vertical meridian toward the fixation point for 0.25° at the speed of 5 °/s: collinear motion (CM) condition (Figure 1C and G), and in a different and final condition, for the same extent and at the same speed, the Flank moved orthogonally to the right of the vertical meridian: Orthogonal Motion (OM) condition (Figure 1D and H). In all conditions: ST, CM, and OM, the target in the target-present trials was presented, randomly, with six Michelson contrasts with respect to the background: 0.100, 0.107, 0.123, 0.140, 0.158, and 0.175 for 30 times per contrast in 8 blocks for a total of 240 trials per condition. In addition, target-absent trials were presented 18 times in the 8 blocks of ST (Figure 1E and F), CM (Figure 1E and G), and OM (Figure 1E and H) conditions. The three observers started the experimental session with a different condition and had the three conditions in a different order throughout the experimental session.

### Experiment 2

**Figure 2.**
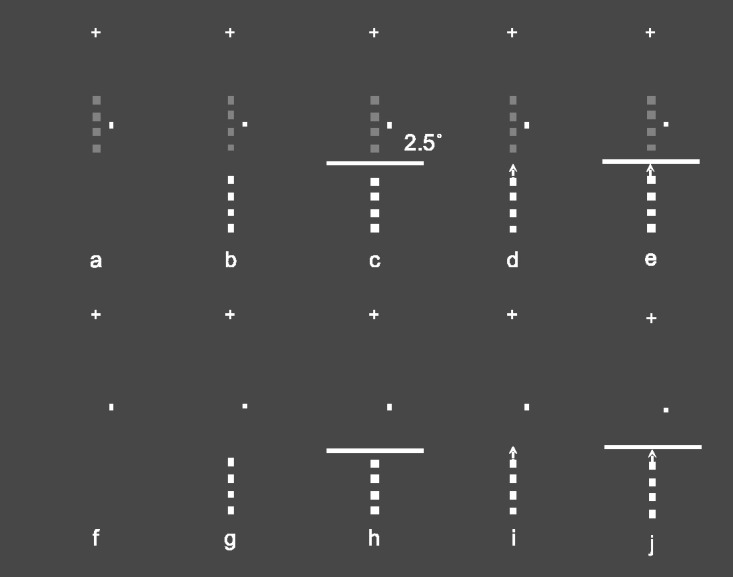
Stimuli in Experiment 2. Target-present (A) and target-absent (F) trials used as baseline to evaluate the context effect. Target-present trials (B, C, D, and E) and target-absent trials (G, H, I, and J) in the two experimental conditions. In detail: Static (ST) condition: target-present (B and C), target-absent (G and H); Collinear Motion (CM) condition: target-present (D and E), target-absent (I and J). The small white cross above each panel represents the fixation point, and the small rectangle to the right of the Target, the attentional cue denominated Dot, was present in the display at all times.

The trials were presented only in the ST (target-present: Figure 2A to C; target-absent: Figure 2F to H) and CM conditions (target-present: Figure 2A, D, and E; target-absent: Figure 2F, I, and J) as in Experiment 1, but in one third of the target-present trials per condition, the Flank was presented with a 2.5° × 0.03° white motionless horizontal bar (Border), at 0.2° below the last squared element of the target, laying on its major side and centered with respect to the vertical meridian (target-present: Figure 2C and E, target-absent: Figure 2H and J).

In the CM condition, the collinearly moving flank moved for 0.25° toward the Border without overlapping it (see Figure 2E and J). As in Experiment 1, the target, with and without the Border (see Figure 2A to E), was presented, randomly, with six Michelson contrasts with respect to the background. Pilot measurements indicated that the presence of a bright Border required a reduction of the contrasts to enhance the CE effect in all conditions. The contrasts were slightly lower than those in Experiment 1 as follows: 0.096, 0.115, 0.133, 0.149, 0.167, and 0.188 and were presented 20 times in 8 blocks per condition for a total of 160 trials. In addition, target-absent trials, as the Targets with and without the Border (see Figure 2F to J), were presented 16 times in the 8 blocks of ST and CM conditions. One observer started the experimental session with the ST condition and the other two with the CM condition.

### Experiment 3

**Figure 3.**
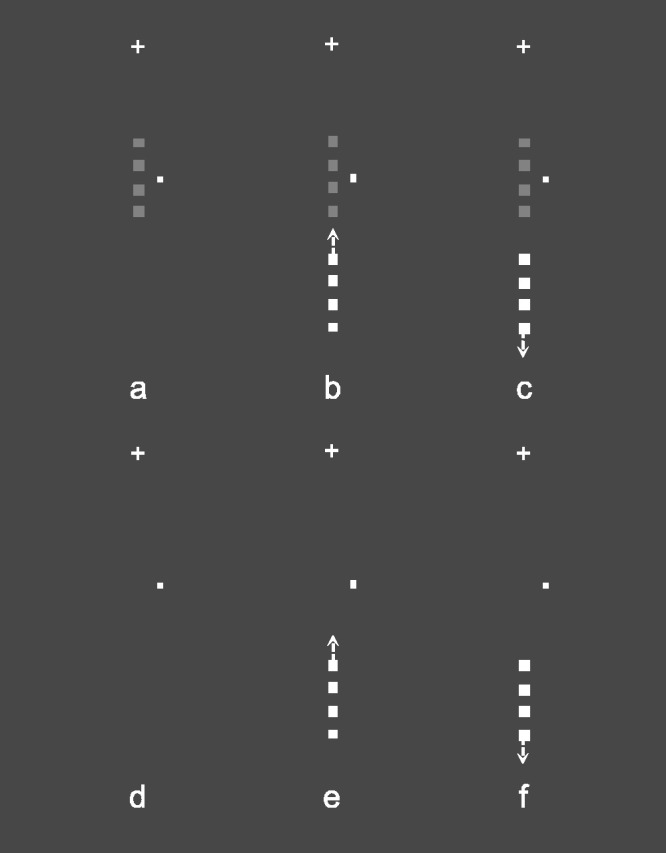
Stimuli in Experiment 3. Target-present (A) and target-absent (D) trials used as baseline to evaluate the context effect. Target-present trials (B and C) and target-absent trials (E and F) in the two experimental conditions. In detail: Collinear Motion-Toward (CM-Toward) condition: target-present (B), target-absent (E); Collinear Motion-Away (CM-Away) condition: target-present (C), target-absent (F). The small white cross above each panel represents the fixation point, and the small rectangle to the right of the Target, the attentional cue denominated Dot, was present in the display at all times.

Two experimental conditions were tested in this experiment: CM with the Flank collinearly moving toward the target as in the previous two experiments, called CM-Toward (see Figure 3B), and CM with the Flank collinearly moving away from the target (downward), called CM-Away (see Figure 3C); Toward and Away refer to the direction of motion of the Flank with respect to the position of the Target in the Target+Flank trials. The speed and motion extent of the Flank were the same as in the previous two experiments.

In the two conditions—CM-Toward and CM-Away, the target, in the Target and Target+Flank trials (Figure 3A to C), was presented with six Michelson contrasts with respect to the background as those used in Experiment 1: 0.100, 0.107, 0.123, 0.140, 0.158, and 0.175 for 30 times per contrast in 8 blocks per condition for a total of 240 trials. In addition, target-absent trials (Figure 3D to F) were presented 18 times in the 8 blocks of CM-Toward and CM-Away conditions. One observer started the experimental session with the CM-Toward condition and the other two with the CM-Away condition.

The percentage of yes responses as to the presence of the target in each target-present and target-absent trial of all experimental conditions in the three experiments was recorded to compute the detection threshold at each contrast. Guessing was compensated by calculating a false-positive rate (*fp*) from the number of false alarms. This *fp* was then used to adjust the percentage of correct responses (*p*) for each contrast according to the formula: *p’= (p–fp)/(1–fp)*, where *p’* was the true percentage of correct responses (Kapadia et al., 1995). A psychometric function was ﬁtted to the *p’* of the six contrasts for each condition using Probit (Finney, 2009) to estimate the contrast required to produce 75% correct detection of the Target. The CE yielded by the Flank for the different experimental conditions was computed as the difference in contrast threshold with and without the ﬂank divided by the contrast threshold without the ﬂank (i.e., the change in detection threshold due to the presence of the ﬂank). The data from the eight blocks were grouped so as to provide three data points for each observer. Paired sample *t* tests were then performed onto the nine data points in each experimental condition in the comparisons indicated in the Results section for each experiment.

## Results

### 

### Experiment 1

**Figure 4.**
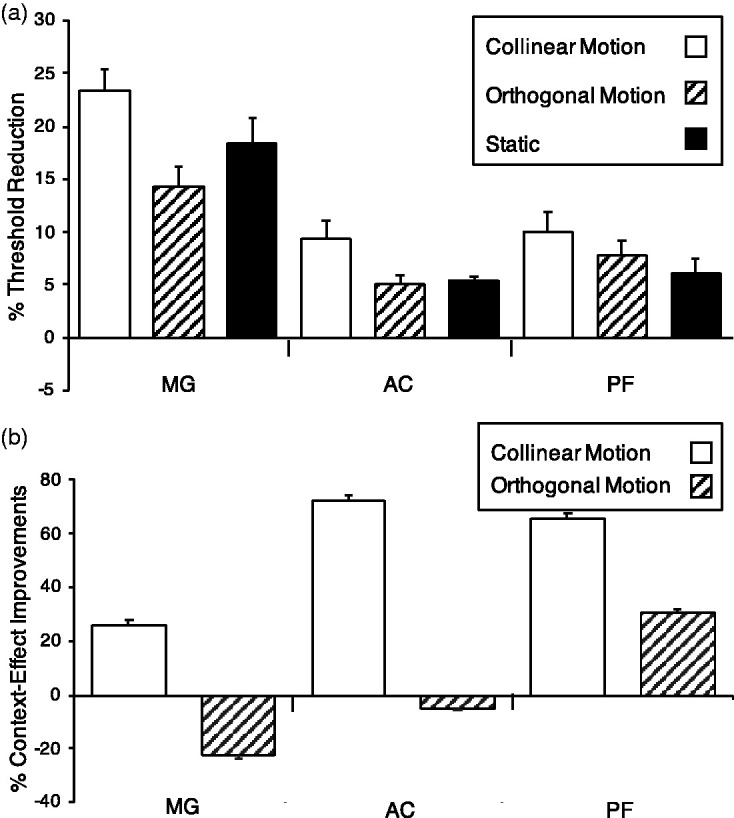
Results of Experiment 1. Panel A shows, in percentage, the threshold reduction for the Target+Flank with respect to the Target only (context effect) in the three experimental conditions: Collinear Motion (CM; white), Orthogonal Motion (OM; black-and-white upward diagonal), and Static (ST; black), for the three observers: MG, AC, and PF. Panel B shows the context effect improvements, in percentage, of the two dynamic conditions CM (white) and OM (black-and-white upward diagonal) with respect to the ST condition for the three observers MG, AC, and PF. Error bars represent the standard error of the mean (SEM).

The presence of the flank collinearly aligned to the target lowered the detection threshold in all conditions for the three observers (Figure 4A) as it was expected from previous studies (Kapadia et al., 1995; Polat & Sagi, 1993, 1994). Mean threshold reductions in percentages (standard error of the mean (SEM)) were as follows: ST = 11.7 (2.5), CM = 16.4 (2.9), OM = 9.9 (1.7).

The novel result of the present study was that the CM condition showed, in all observers, the largest change in detection due to the presence of the collinearly moving flank with respect to the OM and ST conditions. Paired *t* tests confirmed this novel result in that the CM threshold reduction was significantly larger than those in ST and OM conditions—CM versus ST, *t*(8)=3.28, *p* = .011; CM versus OM, *t*(8)=4.63, *p* = .002—while the latter two conditions did not differ from each other—ST versus OM, *t*(8)=1.24, *p* = .249. To better evaluate the difference of the two motion conditions, namely CM and OM, the CE in the ST condition was considered the baseline, and the changes in the other two conditions were calculated with respect to it (Figure 4B). The collinear motion greatly affected the CE in the CM condition with threshold reductions ranging from 26% to more than 70% with respect to the ST condition. With respect to the ST condition, all observers showed the largest threshold reduction in the CM condition. While observer PF had an intermediate threshold reduction in the OM condition, the two other observers showed an opposite-direction CE leading to negative percent improvement values. Therefore, for these two observers, target detection thresholds in the OM condition were larger than in the ST condition. Thus, enhanced detection of the target was not due to motion per se but to the collinear motion of the ﬂank with respect to the target.

### Experiment 2

**Figure 5.**
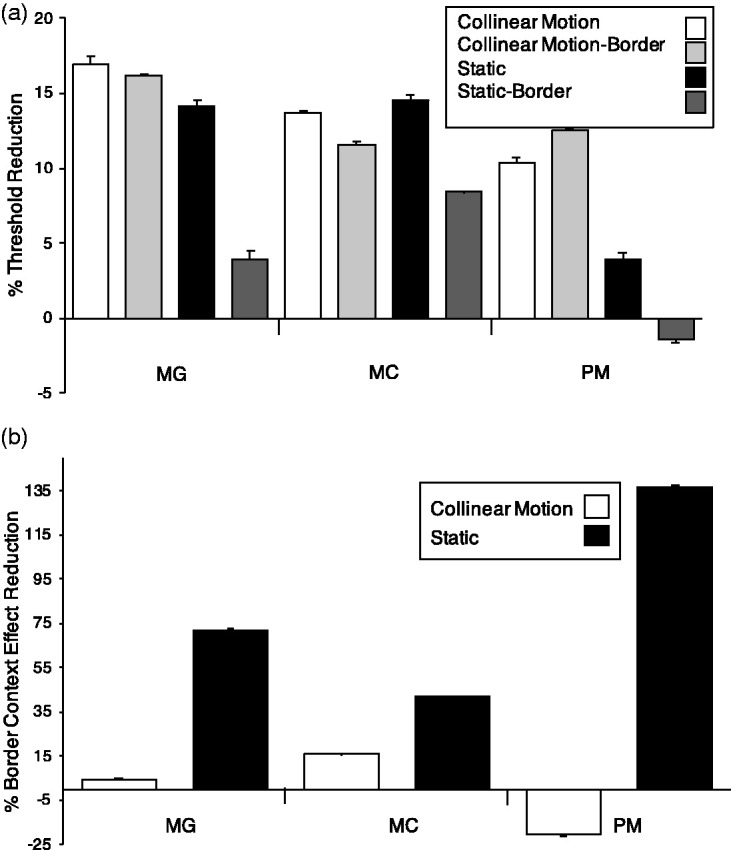
Results of Experiment 2. Panel A shows, in percentage, the threshold reduction of the context effect in the four experimental conditions: Collinear Motion (CM; white), Collinear Motion + Border (light gray), Static (ST; black), and Static + Border (dark gray) for the three observers: MG, MC, and PM. Panel B shows, in percentage, the relative reduction of the context effect due to the presence of the Border in the CM (white) and in the ST (black) conditions for the three observers: MG, MC, and PM. Error bars represent the standard error of the mean (SEM).

The CE showed in Experiment 1 was replicated in Experiment 2 in that the collinear flank lowered the detection threshold of the target in the CM and ST conditions up to 17% (white bars with respect to black bars in Figure 5A). Although the simultaneous presence of the Border greatly reduced the CE in the ST condition—mean (SEM) = 8.3 (1.8) compare black bars with dark gray bars in Figure 5A—in the CM, the reduction was, if anything, negligible—mean (SEM) = 1.3(1.4)—as if the collinear moving flank could overcome the interruption of the collinear path (compare white bars with light gray bars in Figure 5A). Paired *t* tests confirmed this novel result in that the CE reduction in ST condition due to the Border was significantly larger than that in CM condition—ST versus ST+Border, *t*(8)=4.56, *p* = .002; CM versus CM+Border, *t*(8)=0.91, *p* = .389. The graph in Figure 5B represents, in percentages with respect to the condition without the Border, the reduction of the CE caused by the Border in the CM and ST conditions (higher values represent large reduction of the CE, which means that the Border stops the CE). This reduction is clearly larger in the ST with respect to the CM condition for the three observers. For the observer PM (rightmost two bars, Figure 5B), the presence of the Border in the CM condition even constituted an advantage showed by the negative values of the white bar in the graph. As a possible interpretation of this result, CM might assign an additional segregation in depth of the target+flank ensemble with respect to the Border, preventing the latter from interrupting the collinear path of target+flank ensemble and the propagation of the CE from the Flank to the Target. By the same token, the rightmost black bar in Figure 5B exceeding the 100% value, representing the reduction in the ST condition for the observer PM, is due to the negative value in the ST-Border condition indicated in Figure 5A (rightmost dark gray bar).

### Experiment 3

**Figure 6.**
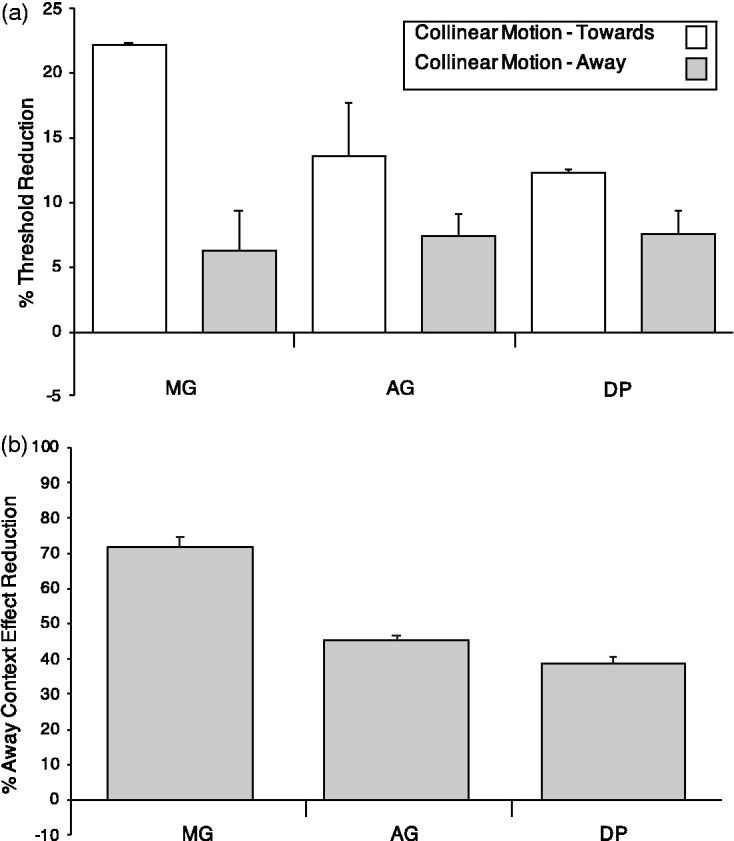
Results of Experiment 3. Panel A shows, in percentage, the threshold reduction of the context effect depending on the direction of collinear motion of the flank with respect to the target in the Collinear Motion-Toward and Collinear Motion-Away conditions for the three observers: MG, MC, and PM. Panel B shows, in percentage, the relative reduction of the context effect in the Collinear Motion-Away condition with respect to the Collinear Motion-Toward condition for the observers: MG, MC, and PM. Error bars represent the standard error of the mean (SEM).

The CE showed in the previous two experiments was replicated in Experiment 3 where the collinearly moving flanks lowered the detection thresholds of the target but the direction of CM had a different effect (Figure 6A). In detail, when the flank moved toward the target along the collinear axis, the CE was as large as 22% (mean (SEM) = 17.4 (1.5), white bars in the figure), while when the flank moved away from the target, the CE was only 6% to 7% (mean (SEM) = 7.6 (1), light gray bars in the figure). Paired *t* tests confirmed this novel result showing that in the CM-Toward condition, the threshold reduction was significantly larger than that in CM-Away condition: CM-Toward versus CM-Away, *t*(8)=4.96, *p* < .001. Figure 6B shows, in percentages with respect the CM-Toward condition, the reduction of the CE when the flank moved, in the CM-Away condition, away from the target. These robust reductions showed that the Away direction not only increased the flank-to-target distance reducing the spreads of activity onto the target, but likely it automatically captured visual spatial attention onto the opposite spatial location with respect to the target.

To better evaluate the different effect of CM direction onto the CE in Figure 6B, clearly in the graph, the CM direction away from the target (CM-Away) yielded a CE smaller, up to 70%, than that produced by the direction toward the target (CM-Toward).

## Discussion

Because it was observed, to my knowledge, the CE has never been produced by a flank collinearly moving toward the target. The parameters manipulated in the attempt to globally describe the effect were, among others, the collinear offset between the target and the flank, the interruption of the collinear axis by a horizontal element, and, finally, the collinear distance between the two elements (Kapadia et al., 1995; Polat & Sagi, 1993, 1994). The experiments of the present study showed a novel dynamic manipulation of these parameters by moving the flank: collinearly or orthogonally with respect to the collinear axis, or collinearly toward a horizontal Border, or, finally, collinearly toward or away from the target. The visual apparent motion, as it was used in this series of experiments, was produced more by the local motion signals than by the global motion of the whole flank (Marr, 1982). By using four squared elements collinearly aligned along the longitudinal axis as flank, there were four moving borders that provided the local motion signals to detect correspondence providing also the direction of motion (Anstis, 1980; Westheimer & Wehrhahn, 1994). As soon as the motion signal was extracted in the visual scene, the spatial arrangement in time of the single elements coherently moving as a whole suggested to higher order visual areas that a particular visual path has become dynamically relevant for the perception of the visual elements in the next future (Verghese & McKee, 2002; Watamaniuk, 2005). Verghese’s study suggested that consistent direction, that is, straight trajectory, of motion is used by the human visual system to enhance detectability of objects in noisy environments. This type of motion is a perceptual cue so effective that it can increase sensitivity to the relevant elements, moving in straight trajectories, even implicitly, that is, when the first part of a two-segment trajectory, acted as a powerful cue although it was barely visible. Thus, the straight trajectory of motion spreads ahead of the moving element consistently with the cued direction of motion. Moreover, Watamaniuk’s study showed that the influence of a motion trajectory propagates in space and time after the end of motion. This induced signal is very specific in that the propagation of the signal takes place only in the inducing direction. Therefore, during visible motion, the visual system must build an expected and more likely trajectory of motion of the relevant moving element that propagates in front of the element on the induced direction of motion. An example of this dynamic effect is a train with several cars moving on a railroad: The front sides of the locomotive and of the cars provide the local motion signals. Once extracted, the motion signal is applied to the train as a coherent whole. Given the direction of the moving train, it is possible for the visual system to predict the future position of the train along the path in front of it but not for the path behind the train. Collinear motion thus becomes a powerful attentional cue that in turn helps in perceiving motion as in the line motion illusion (Hikosaka et al., 1993). Such prediction in time allows us to expect a train coming out of a tunnel or getting to a station which is in our view in the visual scene. As a further example, some car producers provided their vehicles with indicators not only with a simple blinking light but with a series of diodes turning on in sequence outward with respect to the center of the car which give the percept of a line growing in a collinear direction. This apparent moving signal is more efficient in conveying the direction that the car will shortly take to following drivers. It does so not because it simply indicates a side of the road, but it elicits the representation of a real movement in the physical world which is best represented by a collinear direction of motion. Therefore, collinear motion appears to be very useful for the detection of moving elongated objects in particular when they are part of an ensemble (target+flank) that might suggest the presence of a border in the scene (Verghese et al., 1999). On the other hand, orthogonal motion of elongated objects is effective as a global motion signal, but it lacks the enhancement of a motion path that would be powerfully activated only by the collinear motion.

Motion correspondence problem was considered by some authors equivalent, for some aspects, to the stereo correspondence problem (Marr, 1982), at least for rigid bodies as were the stimuli in this study. The crucial difference between the two being that the former is in time while the latter is in space. Motion and stereo are represented in the same higher cerebral visual area, namely area MT, where there are units showing preferred direction of motion and units showing preferred depth (Born & Bradley, 2005). This link between the two computational problems might explain why the collinear motion was able, in Experiment 2, to overcome the interruption of the collinear path by the horizontal Border. The collinear motion of the flank toward the Border, as reported by the naïve observers, gave the impression of the flank moving in a different, in particular, a deeper, plane with respect to that including the Border; therefore, the interference of the latter was abolished. Therefore, the collinear motion yielded a segmentation in depth of the target+flank ensemble with respect to the plane containing the Border. The target was pulled to a deeper plane, even though it was motionless, by the propagating CE from the collinearly moving Flank to the target.

As a further evidence of the motion path activated by an elongated object moving collinearly, Experiment 3 indicated that not only the collinear motion enhances the perception of an object lying onto the motion path but the direction of collinear motion counts. Collinear motion toward a relevant object (target) or position along the motion path propagates resources to accomplish cognitive operations of detection and/or discrimination. As the units in primary visual cortex are lined up according to orientation preference to trigger a growing border, they are activated and integrated (Welch et al., 1997) by collinear motion. This activation propagates ahead, preceding the stimulation of a given unit along the motion path, as a wave (Krekelberg & Lappe, 2001). The opposite direction of motion, that is, away from the Target, if anything, although it comprises spatial positions on the collinear motion path, will propagate the activation in the opposite direction with respect to the target, and therefore, it will go undetected. However, one should bear in mind that only the flank moved in all experiments of this study, and the observer’s task was to detect the motionless target. Therefore, the activation of the motion path propagates ahead onto the target yielding the CE by the spatial proximity of the target and the flank.

This CE effect, sensitive to motion direction, likely is implemented in the visual system from the LGN to area MT, and it requires both feedforward and feedback activity. In V1, the activity of connected units with the same orientation sensitivity, implemented in the LRHC plexus (Cass & Spehar, 2005; Gilbert et al., 1996; Kapadia et al., 1995), might get potentiated by a direction of motion collinear with the orientation of the developing border. This sort of elongated receptive field that encompasses a motion path (Michel et al., 2013) reduces the delay of these units by an anticipatory facilitation fed forward along the path (Watamaniuk, 2005). To do that, the direction of motion of the flank must be detected and discriminated in higher visual areas, such as MT, where the direction of motion and the segmentation in depth are quickly detected by analyzing the direct information coming from LGN (Sincich, 2004) and getting back to V1 to modulate the activity of the LRHC plexus via a feedback pathway (Cudeiro & Sillito, 2006; Sillito et al., 2006): Although this explanation is speculative, the results of the present study are compatible with this implementation in the visual system.

## Conclusions

This study showed that a static contextual effect where particular spatial arrangements of visual elements improve the capability of the human visual system to detect objects can be enhanced by the motion of the visual elements surrounding the object to be detected. Crucially, the motion required must be collinear to the longitudinal axis of the object and in the direction toward it. Thus, collinear motion plays an important role in particular visual scenes where motion is the distinctive feature of objects.
